# Supplementary material for: Are carbon emissions trading and green financial instruments synergistic? -Comprehensive quantitative research based on content analysis
Source: PLoS One. 2024 Mar 7;19(3):e0298601. doi: 10.1371/journal.pone.0298601 (PMC10919601; doi:10.1371/journal.pone.0298601)
Supplement: S1 Data — (ZIP) [file pone.0298601.s001.zip › Data/地级市Z_F工作报告.zip!/║■▒▒/╛ú├┼╩╨/└┤╫╘╣1⁄2╓┌║┼í╛┬φ┐╦╩2╛▌═°í┐_2013.docx]

各位代表：

　　现在，我代表市人民政府向大会报告工作，请予审议，并请各位政协委员和列席人员提出意见。

　　一、2012年工作回顾

　　2012年，在省委、省政府和市委的坚强领导下，在市人大和市政协监督支持下，我们深入贯彻落实科学发展观，高举中国农谷旗帜，按照稳中求进工作总基调，始终坚持“两抓、两建、两重、两促”，较好地应对了宏观经济下行压力，克服重重困难和挑战，完成了市八届人大一次会议确定的工作任务，迈上了“千亿经济总量、百亿财政收入”新台阶。全年实现地区生产总值1085.26亿元，增长12.2%；规模以上工业增加值560.12亿元，增长16.9%；全社会固定资产投资794.40亿元，增长34.5%；社会消费品零售总额347.05亿元，增长16.1%；居民消费价格涨幅3.4%；预计完成财政总收入107亿元，增长12%，其中，地方一般预算收入49亿元，增长24.4%；实际外商直接投资21656万美元，增长15.9%；出口46577万美元，增长21.7%；城镇居民人均可支配收入17678元，增长13.9%；农民人均纯收入9387元，增长13.8%；城镇登记失业率4.2%，人口自然增长率4.45‰，完成省定节能减排目标。

　　一年来，我们主要抓了以下几个方面的工作：

　　（一）着力稳增长。突出投资拉动，全市固定资产投资增幅高于全省平均水平8.1个百分点。大力实施“三千活动”，及时出台帮扶企业共渡难关的“六条措施”，工业生产企稳回升，新增亿元企业100家、净增规模企业100家，规模工业增加值总量居全省第4位，在全省同类城市中领跑。克服多季连旱的严重影响，取得“一抗三保”重大胜利，粮食增产1.28亿斤，实现“九连增”。不断扩大对外开放，设立9个产业招商分局，探索政府办事处与商会秘书处协同招商新机制，共引进市外境内资金660.11亿元，增长113.4%；化工医药及轻工产品出口逆势上扬，同比分别增长18.5%、471.5%。强化要素保障，引进汉口银行设立分行，建成荆门中小企业金融服务网，发行城投债8亿元，全年新增贷款72.98亿元，增长20.0%；投资8.7亿元实施土地整治44万亩，新增耕地2.7万亩。我市作为唯一代表，在全国会议上交流了保护耕地、保障发展的做法和成效。

　　（二）着力壮产业。骨干支柱产业不断发展壮大，化工、农产品加工两大规划千亿产业分别完成产值778亿元、804亿元，建材产业产值达到254亿元，新能源、高端装备、节能环保、通用航空等新兴产业加快发展。国家现代农业示范区建设扎实推进，编制了中国农谷总体发展战略规划、屈家岭考古遗址公园详细规划，推进了核心区、“荆三角”和杨竹流域三个中国农谷示范区规划建设，引进了隆平高科产业科技园、汇源生态产业园、广州奈美国际化妆品产业园等项目，建立了袁隆平、傅廷栋等6个院士工作站。全市新增蓄水能力4亿立方米，新增和改善灌溉面积82万亩。农业综合机械化水平达69%。流转土地承包经营权面积达到50万亩。农民专业合作社基本覆盖全市镇村。新增10亿元龙头企业7家，农产品加工产值与农业总产值之比由2.1：1提升到2.5:1。现代服务业加快发展，中石化荆门销售公司正式成立，星球商业中心、美颂雅庭家具建材城建成投入运营，引进了沃尔玛、苏宁电器等知名商贸连锁企业。再生资源回收利用体系建设成为国家试点。休闲旅游亮点纷呈，“中国农谷•生态荆门”旅游形象片在中央电视台播出，一批旅游项目建设顺利推进，举办了第五届油菜花旅游节，承办了荆门国际乡村旅游论坛、鄂中微旅游博览会、湖北省首届精品菊花展暨荆门市第十九届菊花展、中国（京山）网球节等节会，全市共接待国内外游客1504.11万人次，实现旅游总收入75.33亿元，分别增长36.42%和41.94%。

　　（三）着力抓项目。深入开展“项目建设年”活动，积极推进“项目建设百日大会战”，全市在建亿元以上项目267个，其中新开工173个。荆门石化柴油质量升级改造、天茂碳四制丙烯、大峪口磷复肥技改、金龙泉米糠蛋白等62个亿元以上工业项目建成投产，格林美城市矿产资源循环产业园、华能热电联产、百盟慧谷、广东楚商国际产业城、湖北财富农业科技园、屈家岭农谷创业科技园、市一医南院和农谷大道等项目加快建设。一批重大项目前期工作取得新进展，荆门石化1000万吨扩能改造可研报告已上报中石化初审，环评报告已完成；蒙西铁路（荆门段）可研报告完成评审修编，已开展线路定测工作；漳河机场改扩建项目前期工作进展顺利；老工业基地改造纳入国家调整改造规划范围；荆门石牌核心港区建设工程（一期）完成可研报告及初步设计审批工作；葛洲坝水泥有限公司重组工作已完成，工厂异地技改搬迁工程已启动。谋划储备了一批重大项目，其中已开展前期工作且投资10亿元以上的项目30个，总投资1600多亿元。

　　（四）着力抓园区。出台了“一核六片十五园”综合考评办法，编制完成了总体规划、产业规划和主要专项规划，完成固定资产投资310亿元，占全市投资总额的39%，增长55%；实现规模以上工业总产值1100亿元，占全市总量的55%，增长23%，拉动全市工业经济增长11个百分点。荆门高新区创建国家高新区工作通过科技部验收，格林美南区塑木型材园和西区废渣废泥处理、金龙非织布、宜洋汽车后市场等项目建成投产。荆门化工循环产业园和屈家岭经济开发区升级为省级开发区，钟祥经济开发区被批准为省级高新区，京山、沙洋经济开发区和东宝工业园发展质效不断提升。

　　（五）着力建新城。城市总体规划修编通过专家评审，漳河新区总体建设方案获省政府批复，建立漳河新区规划体系，完成14项规划编制工作。双喜新社区（一期）交付使用，漳河大道完成快车道稳定层施工，政务中心、国华人寿暨天平车险全国电话营销中心等项目开工建设。中心城区管道安全取水工程按期建成，竹皮河城区段综合整治已启动，30条小街小巷改造顺利完工。被评为“湖北省森林城市”。市区城管新体制操作到位，网格化社会管理和数字城管系统建成并试运行；推进城管三年行动计划，一年常态化目标基本实现。城建集团组建运营。各县（市、区）城市新区建设全面启动，镇域小城市及各类重点小城镇加快发展，41个省级示范宜居村庄建设进展顺利，两年累计完成农村危房改造7351户。全市新增转移人口近6万人，城镇化率超过50%。

　　（六）着力惠民生。全力办好10件实事。全市城镇新增就业4.8万人。扶持创业5870人，带动就业2.35万人，被授予“全国创业先进城市”。城乡居民养老保险综合参保率达92%，全市共有30万名60周岁以上老人按月领取养老金。基本药物实现“零差率、全覆盖”，平均药价下降28.5%。新农合参合率达99.5%，人均筹资标准由230元提高到300元。城乡居民电子健康档案建档率达75%。全市7.9万名退休人员不再缴费，终身享受医疗保险待遇。城乡低保再次提标，分别发放医疗救助金、临时救助金2384万元、1614万元。新开工建设各类保障房1.47万套，竣工9496套，新增廉租房租赁补贴2039户。落实义务教育经费保障机制改革资金1.68亿元，对学生校外托管机构进行规范管理，新增校车196辆，钟祥、东宝和掇刀义务教育均衡发展通过省级验收，普通高考再创佳绩。新、改、扩建30所乡镇公办中心幼儿园，资助159名贫困大学新生入学。举办了市第六届运动会和农家乐杯文艺比赛、社区文化节等群众性文化体育活动，承办了全国航空航天模型锦标赛和现代五项少年锦标赛，建成村文化活动中心和文化中心户52个、农家书屋1633家、社区图书室10家，提档升级村级综合服务社300家。组建了荆门广播电视台(集团)，广播电视发射塔迁建工程开工建设。完成国省道大修74.9公里，建设县乡道122公里、通村公路447公里，荆新线一级公路荆门至沙洋段建成通车。对城区8家菜市场进行了改造升级，解决了10万人的饮水安全问题，办理1.3万余件法律援助案件。全面加强信访工作，认真排查化解矛盾纠纷，确保了社会和谐稳定。深入开展复式执法，“两抢一盗”刑事警情持续下降。安全生产形势保持平稳，食品药品安全监管工作不断加强。国防动员和双拥共建深入开展，出生人口性别比和低生育水平保持稳定，扶贫开发、援疆、援恩工作成效明显，人防、防震减灾、消防、应急救援、气象工作积极推进，国家安全、保密、机关事务、地方志、民族宗教、外事侨务、移民、档案、仲裁工作得到加强，科普、妇女、儿童、老龄、慈善、残疾人工作取得新进步。

　　（七）着力转作风。认真开展“喜迎十八大，争创新业绩”主题实践活动。积极开展千名干部进企业和万名干部进社区、进万村挖万塘、洁万家活动。扎实开展整治“庸懒散”专项活动，不断深化勤政廉政建设。建立健全城建例会和县（市、区）长讲规划制度，不断提升决策科学化、民主化水平。共办理人大建议145件、政协提案365件，见面率、回复率100%，满意率98%以上。再造审批流程，审批提速27%，免收行政审批费2000余万元，城乡四级行政便民服务网络覆盖率99.6%，成立了市电子政务办，“四最四零”行政服务品牌进一步彰显。对政府规范性文件进行了清理，废除95件。行政负责人按规定出庭应诉率达到93%。加大行政监察力度，发挥审计监督作用，开展工程建设、土地执法及医疗、教育、涉农等领域专项整治，严肃查处各类违纪违法案件。

　　上述成绩的取得，凝聚着全市人民心血和汗水，得益于社会各界鼎力支持。在此，我代表市人民政府，向全市人民，向人大代表、政协委员，向各民主党派、工商联、无党派人士和人民团体，向驻荆部队、武警官兵、政法干警和中省垂管单位，向所有情系荆门的各界朋友和荆门籍在外人士，表示衷心的感谢并致以崇高的敬意!

　　在报告工作成效的同时，我们也清醒地看到，我市经济社会发展依然存在不少困难和问题，主要表现在：受宏观环境影响，地区生产总值、规模工业增加值和社会消费品零售总额增幅低于年度预期目标；经济结构性矛盾比较突出，环境容量严重不足，自主创新能力亟待提高；扩大就业难和企业用工难矛盾并存，农民持续增收压力仍然较大；化解社会矛盾、维护社会稳定的任务较重；有些工作落实不够，形式主义的问题不同程度地存在；部分干部改革开放意识有待加强，办事效率有待提高。对此，我们要采取有效措施，认真加以解决。

　　二、2013年工作任务

　　各位代表，党的十八大报告明确提出到2020年全面建成小康社会，国内生产总值和城乡居民收入翻一番，省委、省政府提出全省提前到2017年实现这一目标，市第七次党代会提出我市到2016年全面建成小康社会。对照国家全面建成小康社会6类23项指标，我市2011年全面小康实现程度为80%，有9项指标提前实现，有9项指标可在2016年达到或超过，有5项指标难度较大，必须通过艰苦努力才能完成。我们一定要认真贯彻落实党的十八大精神，坚定不移谋发展，着眼长远转方式，联系实际抓重点，统筹兼顾重协调，毫不懈怠惠民生，一以贯之促和谐，力争提前全面建成小康社会！

　　根据市委七届四次全会精神，今年政府工作的总体要求是：全面实施中国农谷战略，紧紧围绕提前全面建成小康社会和综合实力走在全省同类城市前列两大目标，继续实施经济总量倍增、能耗排放和农村人口减量及社会保障覆盖“三大计划”，深入推进工业化、信息化、城镇化、农业现代化“四化”同步发展，努力实现有质量的稳定增长、可持续的全面发展，加快建设“四个荆门”，争当全省科学发展、跨越式发展排头兵。

　　全年经济社会发展的主要预期目标是：地区生产总值增长12%，规模以上工业增加值增长17%，全社会固定资产投资增长25%，社会消费品零售总额增长15%，居民消费价格涨幅控制在4.0%以内，财政总收入增长11%，地方公共财政预算收入增长14%，出口总额增长10%，实际外商直接投资增长12%，城乡居民收入增长12%，城镇登记失业率控制在4.5%以内，人口自然增长率稳定在5.0‰以内，节能减排完成省定目标。

　　重点抓好以下工作：

　　（一）扎实推进新型工业化

　　培育壮大支柱产业。开工建设华工科技产业园、中集安瑞科二期、LED民用照明灯具、荆华铝业工业园、晋煤金楚磷酸一铵、腾宝建材等项目，加快推进荆门石化1000万吨扩能改造、华能热电联产、格林美城市矿产资源循环产业园（东区）、百立恒化工新材料、海鸥卫浴工业园、广东楚商国际产业城、腾邦化工聚苯乙烯等项目，力争明达玻璃、捷力通新材料、力拓阀业、中一钢构（一期）、渝楚化工异辛烷（一期）、五三柒零紧密纺等项目建成投产。继续实施“千百十”工程，力争化工、农产品加工产业规模向千亿迈进，进一步做大做强建材、机电、轻纺等传统产业，不断提高新能源、高端装备、节能环保、通用航空等新兴产业竞争力。狠抓水泥、化肥、煤炭开采和粮油加工企业的兼并、联合及战略性重组，淘汰落后产能，提升产业层次。推动新洋丰、格林美、重组并搬迁改造后的葛洲坝水泥有限公司等10家企业加快形成百亿元规模，支持福耀玻璃、京山轻机等企业尽快超50亿元规模，培育一批销售收入过10亿元的企业，力争新增规模工业企业100家。

　　加强工业园区建设。围绕“十二五”末园区总量4000亿元目标，细化“一核六片十五园”年度目标和推进措施。按照市场化、业主化、多元化原则，创新投入机制，配套完善园区基础设施。探索推进园中园、飞地经济、重点工业集中区等模式，力争各重点园区规模工业总产值超过1400亿元。倾力打造荆门高新区、化工循环产业园两大千亿园区，推动京山、沙洋、钟祥、东宝、屈家岭等园区竞相发展，确保荆门高新区成功创建国家高新区。

　　提高自主创新能力。加快院士工作站以及省级以上重点实验室、工程技术中心和检测中心建设。新增高新技术企业15家，高新技术产业增加值占生产总值的比重达到8.0%。实施“质量兴市”、“品牌兴市”战略，保护、培育一批精品名牌。充分发挥人才支撑作用，注重引进、培养现代金融和城市规划等亟需人才，尽快解决发展中重点人才瓶颈制约问题。

　　大力发展循环经济。探索生态发展模式，完善再生资源回收利用体系，推进废物循环利用、资源综合利用，力争格林美城市矿产资源循环产业园创建国家城市矿产示范基地，农作物秸秆综合利用纳入国家“双百”示范基地。加强资源节约和管理，引导企业开发和应用节能技术，推进节约型机关建设，倡导居民绿色消费。全年组织实施重点节能减排、循环经济项目200个以上，完成投资150亿元。

　　（二）扎实推进农业现代化

　　打造中国农谷“三农特区”。高水平编制完成三个中国农谷示范区规划，深入推进国家现代农业示范区建设。在农谷核心区，以屈家岭管理区为重点，加快京山石龙新农村示范片、太子山中华文化创作交流基地及屈家岭风情小镇、隆平高科种业基地、院士村、农谷农科院等项目建设，提高影响力。在“荆三角”区域，规划推进特色城镇带建设，完成土地整治70万亩，高标准建设百万亩有机稻和双低油菜板块基地，组建全国第1家农机合作社联盟，争创全省现代农业样板区。在杨竹流域，重点建设好东宝牌楼镇“2+6”、钟祥石牌镇“1+5”新型社区，打造全市生态文明新农村示范区。

　　构建现代农业产业体系。坚持试点示范，助推大宗农产品生产向科技要产量、要效益、要生态，力争粮食实现“十连增”，总产新增1亿斤。建设城郊设施蔬菜基地和汉江流域百里蔬菜产业长廊，播种面积达到85万亩。打造“百亿畜禽产业”，新增标准化示范养殖场100个。推广高效渔业养殖模式，创建国家现代渔业示范区。加快龙头企业整合重组，推动国宝、洪森、宝源、广源和兴农等龙头企业向百亿企业发展，新增10亿元企业3个、50亿元企业1个，农产品加工产值与农业总产值之比达到2.8：1。创新农业生产经营体制机制，培育新型经营主体，提升农业专业合作社水平。高标准发展城郊农业，加快发展花卉苗木产业、观光农业和休闲渔业，大力发展以农机、农技等为重点的农村新兴服务业。

　　改善农村生产生活条件。落实国家强农、惠农、富农政策，建立稳定的“三农”投入增长机制。实施防洪保安和抗旱水网工程，硬化末级渠道500公里，除险加固100座小（二）型病险水库。健全农技推广服务体系，重点引进水稻“斜插法”，推广曹武水稻生产全程机械化、五洋油菜精量直播及机收秸秆还田新技术、新模式，建设全国一流农用航空站，力争农业综合机械化水平超过70%。实行最严格的耕地保护制度，继续实施高产农田建设和低丘岗地改造工程。加强气象防灾减灾能力建设，适应气候变化，优化种植结构。继续组织实施农村清洁能源入户工程，建设大中型沼气工程4处，新增清洁能源1.5万户。扎实开展“万名干部进万村洁万家”活动，探索建立农村生活垃圾“户分类、村收集、镇清运、县处理”新机制，基本消除公共卫生死角。

　　（三）扎实推进新型城镇化

　　推动鄂中城市群协调发展。大力实施“1331”城镇发展战略，围绕中心城区“一心四城”空间布局，加快建设100万人口、100平方公里的区域性大城市。支持京山县创建全省“两型”社会示范区和国家生态县、沙洋县建设国家现代农业示范区、钟祥市建设产城融合发展示范区，支持东宝区建设“四化”同步发展先行区、掇刀区建设全省城乡一体化示范区，支持漳河新区建设城乡互化发展先行区、屈家岭管理区建设中国农谷核心区、荆门高新区建设生态科技新城。以胡集、后港、宋河、易家岭等镇域小城市和中心镇、特色镇为重点，深化行政管理、投融资、公共服务等体制改革，进一步完善城镇功能，带动周边乡镇组团式发展和新农村建设。

　　高位推进城市新区建设。健全漳河新区规划体系，完成漳河大道建设，启动西外环和四横四纵主干路网建设，加快推进政务中心、凤凰湖湿地公园、双仙河、国华人寿暨天平车险、中央商务区等项目，倾力打造荆门新城。完成中心城区东外环规划。全面支持京山温泉新区、沙洋滨江新区、钟祥南湖新区、东宝北城新区、掇刀新区、屈家岭农谷核心区和高新区官堰湖生态科技新城加快发展，支持镇域小城市新区规划和建设。

　　加强和改进城市管理。充分运用社会管理网格化和数字城管平台，发挥城管体制机制创新的新优势，深入实施城管三年行动计划，确保实现两年精细化目标。优先发展公共交通，建设城市慢行系统，注重地下空间复合利用，继续开展交通综合整治，有效缓解中心城区区域性时段性交通拥堵。实施市政道路、园林绿化、环卫设施建设管理工程，不断完善小街小巷市政基础设施。继续开展市容环境美好示范路创建活动。建立拆违控违长效机制，全面完成违法建设分类处置工作，确保中心城区违法建设零增长。

　　全面推进“两转四集中三同步”。加快生产、生活、生态融合，深入推进城乡一体化，实现城乡互化发展。坚持土地整治与土地流转、迁村腾地与农民转移、项目建设与政策整合同步推进，引导土地规范化流转，吸引社会资本、技术等要素向农业聚集，力争农村土地流转面积增加20%以上。突破农民在进城落户、技能培训、住房保障、社会保险、子女入学等方面的体制机制障碍，促进农民向城镇转移。完成100个农村新社区规划，抓好周河、新桥、长岗等一批新社区试点示范建设。坚持规划到户、培训到人，力争转移农村人口10万人左右，提高城镇化率3个百分点。

　　实施重大基础设施项目。积极推进蒙西铁路（荆门段）、荆沙铁路改造开通动车组等铁路项目。抓好荆门港石牌港区、沙洋港区、汉江航道综合整治等项目建设。启动钟祥汉江公路二桥工程，完成农谷大道、荆新线沙洋至潜江段、武荆高速公路京山连接线一级公路等公路项目建设。建成北城区供热管网，力争年内供热。开工建设城区天然气环网。规划建设市政务中心冷热联供系统，促进“两型”社会建设。

　　（四）扎实推进信息化和现代服务业发展

　　促进信息化与经济社会发展深度融合。启动“智慧荆门”建设，加强云计算、物联网、大数据和移动互联网的应用，加快网络升级改造和资源共享，推进电信网、广电网、互联网三网融合。实施工业化与信息化融合示范工程，完善信息技术公共服务平台，力争1-2家企业纳入国家试点示范。推动农业适用信息技术广泛应用，抓好沙洋信息精准农业建设试点，提高全市农村信息化水平。加快新闻媒体信息化建设。通过推进电子政务发展，不断提升服务和监管能力。

　　大力发展旅游业。编制中国农谷乡村旅游规划。加快市游客接待集散服务中心、正中水镇、汇源生态产业园、万亩蓝莓休闲园、屈家岭文化遗址公园等项目建设。扎实开展明显陵景区、绿林山风景区创建国家“5A”级景区和彭墩乡村世界创建国家“4A”级景区工作，打造漳河、马河、仙居乡村游精品线路，引进国际品牌酒店，办好油菜花旅游节等节会，接待国内外游客1800万人次，实现旅游总收入90亿元。

　　做大做强商贸物流业。加快推进京山、钟祥、屈家岭和杨家桥等物流中心建设，抓好漳河新区航空服务等项目，支持东宝建设省级服务业综合改革试点区、掇刀建设省级现代服务业示范区，支持李宁物流园开展第三方物流业务。继续实施“新网工程”，扎实抓好商贸流通企业“小进限”工作。

　　积极发展现代金融业。引进1-2家股份制商业银行在我市设立分支机构，力争2家农村信用联社改制为农村商业银行，积极推进村镇银行县域全覆盖、电话银行村级全覆盖。整顿和规范保险业经营秩序，开展政策性保险试点。发展融资担保业，向市中小企业担保公司增资1.5亿元。引导各类金融机构加大信贷投入，提高信贷比。支持企业债券和中期票据发行，做好中国农谷区域集优债发行工作，加快凯龙、新洋丰等企业上市或借壳上市步伐。

　　有效扩大消费和稳定物价。优化城市综合商业网点布局，引导住宿和餐饮业健康规范发展。支持发展消费信贷，培育律师仲裁、估价经纪等中介机构，大力发展文化、信息、健身、培训、养老、家政、网络购物等新兴消费业态。抓好重要商品产运销衔接，支持蔬菜、猪肉直供超市、社区、学校和企业。促进节会消费，抓好“舌尖上的中国农谷”优质农产品武汉展销、2013年（第三届）年货购物节等活动。大力治理“餐桌污染”，坚决打击商业欺诈、制假售假活动，依法查处违规收费和价格违法行为。

　　（五）扎实推进改革开放

　　深化重点领域改革。加快建立国有企业现代企业制度，发挥其引导投资和撬动市场作用，加强国有经营性和行政性资产管理。切实处理好改制企业遗留问题。逐步完善公共财政体系，积极寻找替代财源，大力培植新兴财源。稳妥推进事业单位分类改革，抓好重点镇行政体制改革试点。深化市艺术剧院改革，做好体育场馆管理全国改革试点工作。逐步建立和统一公共资源交易市场，深入推行电子化招投标。

　　进一步抓好招商引资。大力开展以商招商和产业链招商，面向三个层次“500强”，吸引国内外知名企业设立研发中心、区域总部、生产及物流基地和采购中心。加强与中石化、中航工业等央企对接合作，吸引上下游产业和关联产业整体转移或协同转移。鼓励本土企业通过增资扩能、战略合作等途径，扩大规模，增强实力。完善招商引资目标管理及考核办法，突出重点，注重实效，强化落地。

　　加快外向型经济发展。优化外贸结构，重点扶持骨干外贸企业发展，多元化拓展出口市场，力争出口4.9亿美元以上。鼓励扶持优势企业“走出去”布点经营或开展海外投资业务。扩大对外劳务合作，培育全省重点外派劳务基地品牌。积极参与中三角城市群建设，加强与国内外友好城市的交流合作。

　　（六）扎实推进幸福美好家园建设

　　提高就业创业质量，让人民群众体面工作。一是推进自主创业。落实创业补贴和小额担保贷款等优惠政策，筹建小额担保贷款服务中心，建设创业孵化基地群，支持百盟慧谷、聚盛国际等创业基地发展，催生小微企业1000家以上。二是确保充分就业。建设劳动力资源库和岗位信息平台，促进城乡新增劳动力、大中专毕业生顺利就业。三是坚持依法维权。力争纳入用工备案管理的企业达到50%，劳动人事争议调解结案率保持在70%以上。

　　提升社会保障水平，让人民群众减少后顾之忧。一是减少养老之忧。巩固城乡居民社会养老保险工作，确保参保率、续保率稳定在98%以上。在中心城区配套建设10个社区养老服务场所，扩大居家养老服务试点覆盖面，支持社会力量兴办养老服务业。高度重视老龄事业发展，建成省级示范老年大学。二是减少看病之忧。将新农合人均筹资提高到340元，改革新农合支付方式。实行城镇居民、职工基本医疗保险门诊统筹，启动居民医保大病保险，推进单病种限费。实施基本药物省级集中招标采购和零差率销售政策，逐步在二级以上医疗机构配备使用基本药物。完成市一医南院项目主体工程，稳步推进县级公立医院综合改革，办好县级人民医院、中医院和妇幼保健院，提升乡镇卫生院服务能力，支持一批较大规模非公立医疗机构规范发展。大力推进村卫生室标准化建设，力争达标率90%以上。健全中心城区社区卫生服务体系，确保全覆盖。三是减少住房之忧。继续抓好保障性住房建设管理和房地产市场宏观调控，健全多元化住房保障体系，完善住房公积金制度，让更多的中低收入家庭住有所居。四是减少贫困之忧。构建专项扶贫、行业扶贫和社会扶贫三位一体的工作机制，加快移民脱贫致富步伐。发展慈善事业，倡导社会互助。关爱社会弱势群体，标准化管理城乡低保，对农村五保供养服务机构实行星级管理，加大医疗救助普惠力度，做好流浪乞讨人员救助管理和服务工作。

　　坚持公平与效率并重，让人民群众共享公共事业性服务。一是努力兴办优质教育。深入落实学前教育三年行动计划，继续实施义务教育均衡发展“四大工程”，提升普通高中内涵发展水平。打造特色化职业教育，提高荆门技师学院办学层次和水平。改善特殊教育学校办学条件，建立和完善随班就读支持保障体系。继续加强校外托管机构规范管理。促进校车向农村偏远地区延伸。加快开工建设楚天学校。支持荆楚理工学院政策化债，依托其组建荆门职业技术学院。二是积极推进文化建设。加强社会主义核心价值体系建设，广泛开展精神文明创建。组织开展全民阅读、送戏下乡、社区文化、全民健身等文化体育活动。鼓励文艺精品创作。积极谋划妇女儿童及青少年活动中心项目，实现广播电视“户户通”全覆盖，建设农民健身工程150个、村级文化中心和文化中心户40家。加快组建文化体育产业集团，支持广电、报业和文体三大产业集团做大做强。三是统筹抓好各项事业。实施中心城区社区建设三年行动计划，抓好社区公共服务站建设。坚持计划生育基本国策，促进人口长期均衡发展。开展全国第三次经济普查，完成市二届地方志编纂任务，依法管理宗教事务，继续做好援疆和援恩工作，力争国家安全、保密、对台、外事、侨务、统计、档案、防震、气象等工作再上新台阶。

　　致力建设美好荆门，让人民群众亲近碧水蓝天。一是力求空气更清新。综合治理机动车尾气、扬尘、工业废气、秸秆焚烧等污染，加快建设葛洲坝水泥厂异地技改搬迁工程，改善大气环境质量。二是力求水源更清澈。综合治理竹皮河和长湖流域污染，严格落实漳河三圈保护规划，巩固水库投肥养鱼整治成果。三是力求环保更有效。综合防治化工污染、重金属污染和农业面源污染，加强森林、水系、湿地及水源涵养区、生态脆弱区的环境保护，加大城区周边损坏山体的修复治理和矿山土地复垦力度，积极创建全国环保模范城市、国家卫生城市、国家森林城市和国家生态园林城市，全市人民团结一心，建设我们共同美丽的家园。

　　加强和创新社会管理，让人民群众安居乐业。一是化解社会矛盾。持续开展“六五”普法活动，加大法律援助维权力度。加强流动人口、特殊人群、农村留守家庭以及社会组织的服务管理，抓好虚拟社会动态管理和网络舆情引导。完善群众来信来访处理制度，健全社会矛盾纠纷超前排查、源头化解、夯实基础的长效机制，主动防范和妥善化解社会矛盾。加强应急管理工作，提高保障公共安全和处置突发事件能力。二是狠抓安全生产。强化公共安全体系和企业安全生产基础建设，继续开展食品药品安全先进县（市）创建活动，强化源头监管，杜绝重特大安全事故发生。三是深化平安荆门建设。全力推进“三全巡逻”和“四季问安”活动，开展治安复杂地区和突出治安问题的专项整治，依法打击各类违法犯罪活动。

　　扎实推进国防动员工作，深入开展“双拥”创建，支持驻荆部队建设，不断提高预备役部队、民兵和人民防空建设水平。

　　各位代表，人民政府要时时刻刻想着群众。今年我们将筹资30亿元，着重办好以下民生实事：

　　1、全市新增就业44000人，组织就业培训18000人，扶持创业5000人，带动就业20000人。

　　2、将20种重大疾病纳入新农合保障试点范围，建立城镇居民、职工基本医疗大病高额医疗费用二次补偿机制，取消城镇居民、职工基本医疗保险统筹基金最高支付限额。

　　3、新建保障性住房12320套，新增廉租住房租赁补贴1240户。

　　4、拉通“通村断头路”500公里，新增50台空调公交车，改造、新建50处公交站台站棚。

　　5、解决农村10万人的饮水安全问题，提档升级村级综合服务社300家，建设农民健身工程150个。

　　6、资助100名以上贫困大学生入学，新、改、扩建城区幼儿园30所，建成名泉小学。

　　7、为400名城镇、3100名农村残疾人提供实用技术和职业能力培训，为60户贫困残疾人家庭免费实施无障碍改造，为80户农村贫困残疾人家庭实施危房改造。

　　8、加快整村推进22个贫困村扶贫开发。

　　9、实施中心城区菜市场改造升级，启动10家，完成5家。

　　10、中心城区网格化社会管理和数字化城管系统投入运行，增设450个治安视频探头，建设10处公共无线上网区，免费提供市民使用。

　　（七）扎实推进政府自身建设

　　新的一年，我们将继续围绕打造“民本政府”，更加突出为民、务实、创新、法治、清廉，切实加强政府自身建设。

　　更加突出为民。要始终坚持思想上为了人民、工作上立足人民、作风上服务人民、目标上满足人民，加快转变政府职能，强化公共服务和社会管理，加大政府信息公开力度，像抓经济建设一样抓民生保障，像落实经济指标一样落实民生工程，不断提高群众的满意度和幸福感。

　　更加突出务实。要不折不扣地贯彻落实中央八项规定，加强和改进调查研究，确保领导干部有三分之一的工作时间在基层开展调研，深入了解真实情况，面对面解决实际问题。继续推行“三少三短”新政，不摆花架子、不作表面文章。大力整治“庸懒散奢”等不良风气，狠抓重要决策、重点工作、重大项目的推进落实。力戒虚浮，真心为民，夙夜在公，勤奋工作。

　　更加突出创新。要着力创新行政管理方式，进一步清理、减少和调整行政审批事项，深入推行“四最四零”改革，逐级建立主体明确、责权对称、层级清晰、奖罚分明的责任体系。深入解放思想，注重动手操作，善于用改革的方法、创新的思路破解前进中的难题。

　　更加突出法治。要严格按照法定权限和程序行使权力、履行职责，自觉接受市人大及其常委会的法律监督、工作监督和市政协的民主监督，主动接受社会公众监督和新闻舆论监督，认真听取各民主党派、人民团体和社会各界的意见和建议，及时办理人大代表建议和政协委员提案。

　　更加突出清廉。要严格执行廉洁从政各项规定，强化对重要领域、关键岗位的行政监察和审计监督，坚决查处各类违纪违法案件，始终保持惩治腐败的高压态势，坚决以反腐倡廉的实际成效取信于民。

　　各位代表！推进中国农谷建设，我们已经迈开大步；全面建成小康社会，我们正在加速奔跑。让我们紧密团结在以习近平同志为总书记的党中央周围，全面贯彻落实省委、省政府和市委的决策部署，开拓创新，攻坚克难，践行人民政府为人民群众的庄严承诺，以无愧于时代和人民的新业绩，为提前全面建成小康社会做出新的更大贡献！
